# Silver Nanoparticles from *Hermetia illucens* Biomass Are Antibacterial Against *Pseudomonas aeruginosa* Infection in *Caenorhabditis elegans*

**DOI:** 10.3390/microorganisms13061277

**Published:** 2025-05-30

**Authors:** Alinne Ayech, Gabriela Hollmann, Robson M. Marreiro Gomes, Belisa A. Rodrigues, Vanessa K. Engers, Rafael S. Gonçalves, Sandro Daniel Nornberg, Daniela F. Ramos, Karine Rigon Zimmer, José M. Monserrat

**Affiliations:** 1 Programa de Pós-Graduação em Ciências Fisiológicas (PPGCF), Instituto de Ciências Biológicas (ICB), Universidade Federal do Rio Grande-FURG, Rio Grande 96210-030, RS, Brazil; gabihollmann@gmail.com; 2 Programa de Pós-Graduação em Aquicultura (PPGAq), Instituto de Oceanografia (IO), Universidade Federal do Rio Grande-FURG, Rio Grande 96200-970, RS, Brazil; roobinho_matheus@hotmail.com; 3 Programa de Pós-Graduação em Biociências (PPGBio), Universidade Federal de Ciências da Saúde de Porto Alegre, Porto Alegre 90050-170, RS, Brazil; belisa.rodrigues@ufcspa.edu.br (B.A.R.); vanessa.engers@ufcspa.edu.br (V.K.E.); karinerz@ufcspa.edu.br (K.R.Z.); 4 Nui Insect Science Bioinsumos e Biotecnologia LTDA, Avenue Domingos José de Almeida, 1785, sala 4 i, Areal, Pelotas 96085-470, RS, Brazil; rafaeldasilvagoncalves@gmail.com (R.S.G.); nornberg.sandro@gmail.com (S.D.N.); 5 Programa de Pós-Graduação em Ciências da Saúde (PPGCS), Faculdade de Medicina (FAMED), Universidade Federal do Rio Grande-FURG, Rio Grande 96200-190, RS, Brazil; daniferamos@gmail.com

**Keywords:** nanotechnology, bacterial infection, *P. aeruginosa*, antimicrobial, *C. elegans*, green synthesis, black soldier fly

## Abstract

Silver nanoparticles (AgNPs) are known for their antimicrobial properties, and can be synthesized through various routes. We used both chemical synthesis and green synthesis from the biomass of black soldier larvae (*Hermetia illucens*). To test the antimicrobial potential of these nanoparticles, we employed an in vitro test, with CFU counting, and also used the worm *Caenorhabditis elegans* as an in vivo model. *C. elegans* were infected with *Pseudomonas aeruginosa* and treated with AgNPs from both syntheses. These AgNPs exhibited absorption spectrum peaks around 400 nm and sizes of 8 ± 3.5 (chemical) and 11 ± 4.7 nm (green). *P. aeruginosa*-infected worms, without treatment with AgNPs, achieved 100% mortality within 3 days, while AgNPs-treated worms survived until the end of the experiment, with no statistical differences compared to the non-infected worms of the control group. The results demonstrate that nanoparticles produced from *H. illucens* biomass have antimicrobial capacity, reducing bacterial growth in vitro and being able to protect *C. elegans* from infection by *P. aeruginosa*, similar to those produced by chemical synthesis. However, AgNPs from green synthesis are less harmful to the environment while maintaining their antimicrobial potential.

## 1. Introduction

Antimicrobial resistance according to the World Health Organization (WHO) is one of the main global threats to public health. It is estimated that bacterial antimicrobial resistance was responsible for 1.27 million global deaths in 2019 and contributed to 4.95 million deaths. The indiscriminate use of these antimicrobials in humans, animals and plants has been identified as the major driver of the development of drug resistance in these pathogens [1]. Alongside the rise in antimicrobial resistance, the need for more potent antibiotics has become the key focus of research.

In this context, nanotechnology emerges as an extremely important tool, with silver nanoparticles, a highly effective antimicrobial, serving as a promising alternative with a lower likelihood of generating antimicrobial resistance [2]. Nanotechnology development has focused on metallic nanoparticles due to their unique chemical and mechanical properties. These nanoparticles are easily synthesized through green methods using biological extracts that simultaneously act as reducing and stabilizing agents. For example, extracts from *Moringa oleifera* leaves produce silver nanoparticles (AgNPs) with sizes ranging from 10 to 25 nm and exhibit antimicrobial activity [3]. AgNPs are known for their antibacterial properties and have numerous industrial applications [4]. Capitalizing on the antimicrobial capacity of AgNPs, the pharmaceutical industry is currently introducing products aimed at treating bacterial infections that are resistant to conventional antibiotics [5]. There are different methods to synthesize silver nanoparticles, such as chemical and physical methods, but green synthesis offers significant advantages such as lower toxicity and cost-effectiveness [6].

*Caenorhabditis elegans* (Maupas, 1900) (Rhabditida: Rhabditidae) is a suitable model for studying complex interactions, such as bacterial infections, testing potential antimicrobial treatments, and assessing the possible toxic effects of these treatments. This worm feeds on bacteria [7], making it an excellent model organism to evaluate infectious processes as well as antimicrobial treatments [8]. *Pseudomonas aeruginosa* is a potentially pathogenic bacterium known for its multiple antibiotic resistance mechanisms and a broad range of virulence factors. One promising therapeutic alternative that has shown significant effects against pathogenic bacteria is AgNP [9]. Concentrations of approximately 2.5 µg/mL of AgNPs have demonstrated antimicrobial effects on strains considered resistant to antibiotics, with nanoparticles measuring between 5 and 20 nm, resulting in high bacterial lethality [10].

This study aimed to explore the antimicrobial potential of AgNPs synthesized by green methods using extracts from the black soldier fly larvae, *Hermetia illucens* (L.) (Diptera: Stratiomyidae). Insects such as *H. illucens* have evolved innate immune systems to protect them from pathogens. The larvae of *H. illucens* are capable of feeding on various organic substrates and surviving in extremely harsh environments populated by pathogenic microorganisms. This unique adaptation suggests that the larvae of this species may produce antimicrobial substances to combat infections from microorganisms present in diverse substrates, such as manure and vegetable compost [11]. Specifically, we evaluated the ability of these AgNPs to combat *P. aeruginosa* infection in the nematode *C. elegans*, using survival rates as the primary indicator of effectiveness. The objective of this study was to assess the feasibility of using AgNPs as antibacterial agents with a low risk of promoting bacterial resistance.

## 2. Materials and Methods

### 2.1. Caenorhabditis elegans Maintenance

*C. elegans* N2 Bristol (wild-type) worms were maintained on NGM Petri dishes (nematode growth media; 3.0 g of NaCl/L; 5.0 g of peptone/L; 5.0 mg of cholesterol/L, 1 mmol of CaCl_2_/L; 1 mmol of MgSO_4_/L; 25 mmol of KH_2_PO_4_/L; and 17 g of agar/L diluted in 1 L of autoclaved Milli Q water at pH 6.0) with *Escherichia coli* OP50 (a non-pathogenic strain) as the food source, and then kept at 20 °C in BOD [12].

### 2.2. Pseudomonas aeruginosa

*P. aeruginosa* ATCC 27583 (PA27853) was cultured in Tryptone Soya Broth (TSB) at 37 °C for 24 h and the growth was quantified by optical density (OD620 nm) at 620 nm using a spectrophotometer, adjusting its absorbance to approximately 0.9–1.0.

### 2.3. Chemical Synthesis of AgNPs

The chemical synthesis of AgNPs was based on Josende et al. [13], where the AgNO_3_ solution was reduced by sodium borohydride (NaBH_4_) in the presence of sodium citrate dihydrate. Specifically, 0.059 mmol of AgNO_3_ (10 mg) was mixed with 50 mL of distilled water under strong agitation in an ice bath. Then, 0.31 mmol of sodium citrate (92 mg) was added, and after 10 min, 0.063 mmol of NaBH_4_ (2.4 mg) was poured under agitation for 30 min. The ice was removed, and the mixture was stirred for 30 min at room temperature. The final volume of solution was 59 mL.

### 2.4. Extraction of Hermetia illucens Polyphenols and Green Synthesis of AgNPs

Alkaline hydrolysis to extract polyphenols from *H. illucens* larvae biomass was carried out with KOH 2 M at a temperature of 65 °C for 2:30 h using a solvent mass ratio of 20 mL/g in an ultrasound bath at a working frequency of 40 kHz. The pH of the samples was adjusted to neutral with 12 M HCl and centrifuged for 5 min at 10,000× *g* at 4 °C.

Green synthesis of AgNPs was carried out using the hydrolyzed extract of *H. illucens* for 4 h at 40 °C with a concentration of 2.4 µM of AgNO_3_ and 2.5% of the hydrolyzed extract. In this procedure, the extracted polyphenols act as a reducing agent of ionic silver, also adding to stabilize the nanoparticles in the aqueous media.

Total polyphenols were quantified following the Waterhouse method [14] using gallic acid to obtain a standard curve, and values were expressed in μg of gallic acid equivalents (GAE)/g and DPPH (2,2-difenil-1-picrilhidrazil); radical scavenging followed the protocol proposed by Sicari et al. [15]. The scavenging capacity of the DPPH radical was expressed in μM of Trolox (TE) equivalents/g.

### 2.5. Characterization of AgNPs

AgNPs were analyzed using a Jeol (Tokyo, Japan) JEM-1400 120 keV Transmission Electron Microscope (TEM). TEM images of AgNPs were measured using the free software ImageJ^®^ Version 1.54 (National Institute of Health, Maryland, MD, USA). The zeta potential was measured using an Anton Paar Litesizer DLS 500 (Graz, Austria). High resolution TEM (HRTEM) and selected area electron diffraction (SAED) pattern were performed on a TEM TECNAI G^2^ F20 (Hillsboro, OR, USA), 200 kV for the silver nanoparticles obtained through green synthesis. Prior to measurement, the AgNPs were sonicated for 5 min at 10% in a Bonitech Branson sonicator (Brookfield, CT, USA) [16]. The surface plasmon resonance of the nanoparticles in suspension was evaluated using a UV-Vis spectrophotometer (BEL Photonics UV-M51) (Nagavara, India) in the range of 300–700 nm.

### 2.6. Caenorhabditis elegans Infection Assay

The protocol employed (liquid infection assay) was based on a previous study [17] with modifications. *P. aeruginosa* (PA27853) (active) inoculum in TSB (OD620 nm between 0.9 and 1.0) was incubated first at 37 °C for 24 h, and then at 25 °C for 24 h. *E. coli* OP50 (used as a control, solely as food and not as a pathogen) was cultured in Luria–Bertani broth (LB); the inoculum was adjusted to an absorbance at 620 nm of 1.0, and inactivated by heat. L4 larval stage worms, obtained by a synchronization protocol [18], were washed with M9 buffer [3.0 g Na_2_HPO_4_ (141.89 g·mol^−1^), 1.5 g KH_2_PO_4_ (136.02 g·mol^−1^), 2.5 g NaCl (58.43 g·mol^−1^), 500 µL MgSO_4_ (1 mol·L^−1^) and 500 mL of ultrapure H_2_O]. Next, 100 mL of M9 buffer was added to 100 µL of cholesterol. *P. aeruginosa* active and inactivated *E. coli* OP50 (used as a control) were quantified by measuring their absorbance at 620 nm. In 96-well plates, worms (on average 15 per well) were added into 80 µL of M9 buffer supplemented with cholesterol (5 mg·mL^−1^). Then, the plates were divided in groups with or without the addition of 5-fluorodeoxyuridine (FUdR; 50 µg/mL) to sterilize the animals. In the untreated groups, 5 µL of bacterial inoculum was added into the wells: inactivated *E. coli* OP50 for controls, or *P. aeruginosa* for infection. In the treated groups, in addition to bacterial inoculum, 2.5 µL of AgNPs (obtained from chemical and green synthesis) was added to the wells. Each well received a total volume of 150 µL adjusted with M9 buffer supplemented with cholesterol. The plates were incubated at 25 °C and the animals were monitored for five days, with deaths counted daily. Assays with different experimental groups were conducted simultaneously.

### 2.7. Antimicrobial Effect of Chemically and Green-Sinthesized AgNPs Against P. aeruginosa

The antimicrobial effect was evaluated using Mueller–Hinton Agar (MHB) medium, plated on 90 mm Petri dishes [19]. The plates were filled with this medium and then inoculated with *P. aeruginosa* ATCC PA27583. The inoculum was adjusted to 10^5^ CFU/mL, and diluted 10^6^ times in M9 buffer (the same medium that the *C. elegans* worm received) and 1 µL was pipetted onto the plates. The plates were divided into three groups: (1) plates that received only the bacterial inoculum, (2) plates that received the bacterial inoculum and chemically synthesized AgNPs, and (3) plates that received the bacterial inoculum plus green-synthesized AgNP. The AgNPs from both syntheses were pipetted at a concentration of 2.5 µL·mL^−1^ and spread using a rod, before receiving the bacterial inoculum. After one day of growth, the number of CFU colonies (Colony Forming Units) in each group was counted.

### 2.8. Statistical Analysis

The analyzed data represent the mean of six independent assays, each with three replicates. For the *C. elegans* survival curve, Kaplan–Meier log-rank and Cox regression analyses were used. Differences were considered statistically significant at *p*-values < 0.05.

## 3. Results

### 3.1. Characterization of AgNPs

**Figure 1:**
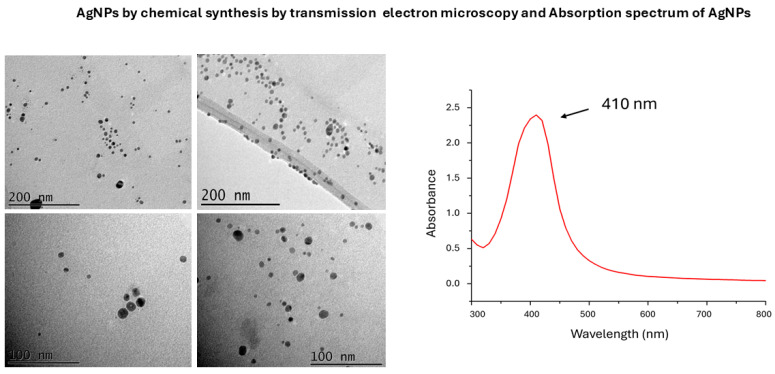
Transmission electron microscopy (TEM) images and UV-visible absorption spectra of silver nanoparticles (AgNPs) chemically synthesized.

**Figure 2:**
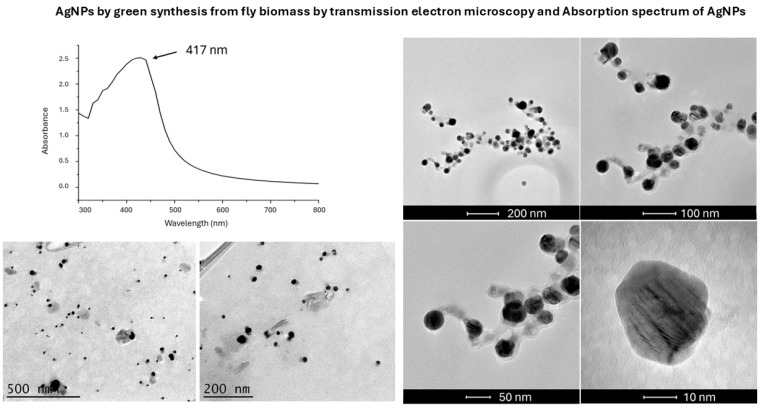
Transmission electron microscopy (TEM) images and UV-visible absorption spectra of silver nanoparticles (AgNPs) synthesized by green synthesis.

**Figure 3:**
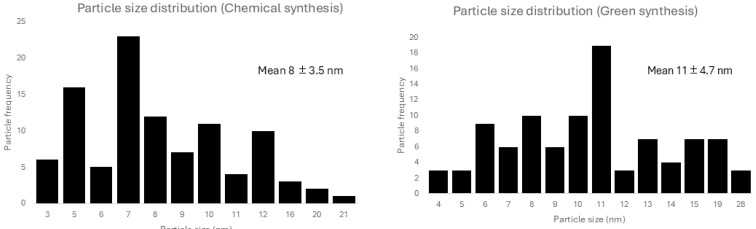
Size distribution of silver nanoparticles from chemical and green synthesis. Mean size and standard deviation is informed for each type of synthesis.

**Figure 4:**
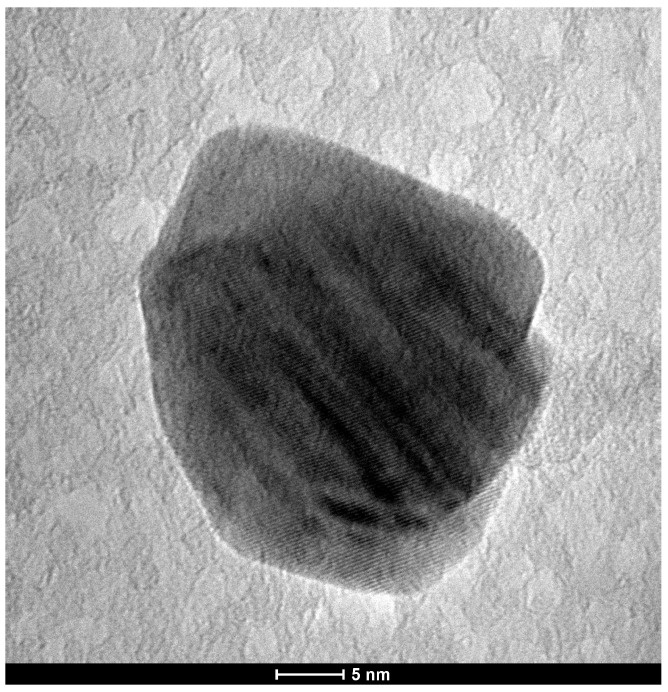
High-resolution transmission electron microscopy (HR-TEM) of green-synthesized silver nanoparticles using *H. illucens* biomass.

TEM analysis showed spherical particles in both chemical and green AgNPs syntheses (Figure 1 and Figure 2). The mean particle sizes were 8 nm and 11 nm (Figure 3) for the chemical and green syntheses, respectively. Both UV-visible absorption spectra of silver nanoparticles (AgNPs) show a peak at approximately 400 nm (chemical synthesis 410 nm and green synthesis 417 nm) (Figure 1 and Figure 2). Electron diffraction analysis of AgNPs obtained via green synthesis is shown in Figure 4.

### 3.2. Total Polyphenols and DPPH Radical Scavenging Activity from Hermetia illucens Extract

The total polyphenol content of the *H. illucens* extract was 54.79 mg GAE/g, with an antioxidant capacity (DPPH) of 69.12 mM TE/g.

### 3.3. Zeta Potential of AgNPs from Chemical and Green Synthesis

The zeta potential for the chemical synthesis was −35 mV, while that for the green synthesis was −22 mV.

### 3.4. Survival of C. elegans to P. aeruginosa

**Figure 5:**
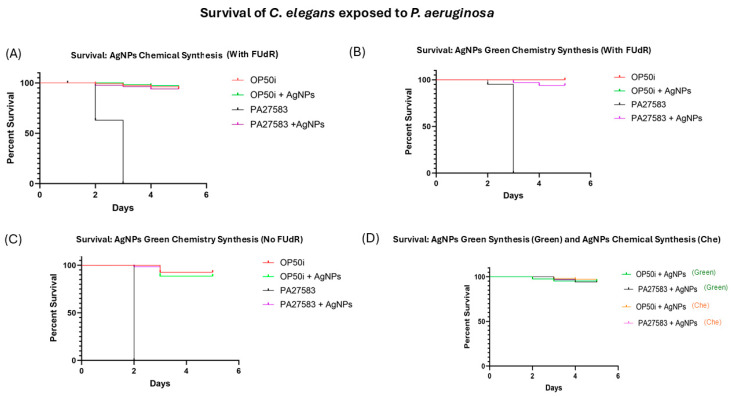
*C. elegans* survival assay. (**A**) Infected worms with *P. aeruginosa* ATCC PA27853 and without treatment reached 100% mortality in 3 days (with FUdR), whereas worms treated with chemically synthetized AgNPs had a survival rate comparable to that of control group (non-infected worms) (*p* > 0.05). (**B**) Infected worms with *P. aeruginosa* ATCC PA27853 and without treatment reached 100% mortality in 3 days (with FUdR), whereas worms treated with green-synthesized AgNPs had a survival rate comparable to that of the control group (non-infected worms) (*p* > 0.05). The red line overlaps with the green line. (**C**) Infected worms with *P. aeruginosa* ATCC PA27853 and without treatment reached 100% mortality in 2 days (no FUdR), whereas worms treated with green-synthesized AgNPs had a survival rate comparable to that of the control group (non-infected worms) (*p* > 0.05). (**D**) Comparison of the survival rates of *C. elegans* treated with chemically synthesized AgNPs and green-synthesized AgNPs using the biomass of a *H. illucens* fly, where treatments and controls showed no statistical difference (*p* > 0.05).

AgNPs treatment improved their survival to values similar to non-infected worms [control group], demonstrating the antibacterial potential of AgNPs and their ability to protect *C. elegans* from *P. aeruginosa* infection (Figure 5). The results showed that PA27583- infected worms reached 100% mortality on the second day [no FUdR] and the third day [with FUdR] of the experiment (Figure 5A,B). Interestingly, worms infected with PA27583 that received FUdR survived longer.

Worms treated with AgNPs obtained by green synthesis using *H. illucens* biomass showed similar results to those treated with chemically synthesized AgNPs (Figure 5A–C). No statistical differences were observed between worms treated with chemically or green-synthetized AgNPs when compared with the control group (*p* > 0.05; Figure 5D).

### 3.5. Antimicrobial Efficacy of AgNPs Against P. aeruginosa

A mean reduction of 88% ± 0.5 CFU was observed in the group that received AgNPs obtained by green synthesis and a mean reduction of 70% ± 0.6 CFU in the group that received chemically synthesized AgNPs. These findings reveal a drastic reduction in *P. aeruginosa* growth in both cases, demonstrating the antibacterial potential of these AgNPs.

## 4. Discussion

As described by [6], there are many advantages in using the green synthesis of nanoparticles, however these advantages are only meaningful if there is no reduction in the antimicrobial capacity of these particles, which we were able to observe in this study. The green synthesis demonstrated antimicrobial capacity comparable to that of the chemical synthesis.

Zeta potentials above ±30 mV indicate more stable solutions, while values below ±20 mV can result in particle aggregation [20], suggesting that chemically synthesized nanoparticles are more stable. From the results, we observe that the chemical synthesis presents a zeta potential of −30 mV, indicating more stable particles with less chance of aggregation, as suggested by [20]. This highlights a gap that needs to be addressed in the search of a more stable AgNPs suspension obtained through green synthesis from *H. illucens* biomass.

The peak obtained in the range of 400 nm to 460 nm confirms the presence of silver nanoparticles [21], as our results indicate.

*H. illucens*, known as the black soldier fly, has already been used in other studies as a source of chitin, using a steam flash explosion to generate chitinous nanoparticles [22]. In our study, we generated AgNPs with antimicrobial capacity through green synthesis from *H. illucens* biomass (Figure 2 and Figure 3). The use of insect biomass in nanotechnology is an emerging field that opens the possibility of developing new products in a sustainable and eco-friendly manner [22]. Notably, AgNPs obtained using *H. illucens* blocked *P. aeruginosa*-induced mortality in *C. elegans* in the same way as those obtained by chemical synthesis (Figure 5D). These results are encouraging because *P. aeruginosa* is a significant cause of healthcare-associated infections and is included in the “critical” category of the World Health Organisation’s (WHO) priority list of bacterial pathogens, for which research and development of new antibiotics are urgently needed [23].

FUdR is an inhibitor of DNA replication used in the treatment of colorectal cancer, but it is also a known blocker of reproduction of *C. elegans* and is widely used in aging research with this worm, as it allows for a synchronous population [24]. One possible explanation for these results found in Figure 5 is that FUdR-exposed worms were more resistant to stress and had an extended health span, as found by Angeli et al. [25].

*C. elegans* is a model widely used in studies of host–pathogen interactions, such as *P. aeruginosa* [26]. The worm is transparent, allowing visualization of the organs through microscopy, which makes it an interesting model for infection [27]. Our results pave the way for further studies, particularly those related to the behavior of nanoparticles inside the worm when infected.

Some studies have shown the role of polyphenols as reducing agents for silver ions, along with how this contributes to the production of nanoparticles [28,29]. Polyphenols have antioxidant properties, and in addition to their interactions with metal ions, they are of great interest in the production of nanoparticles used in medicine and disease treatment [29]. *H. illucens* protein hydrolysates have polyphenols and antioxidant activity, which can be applied to human health, due to their antioxidant and anti-inflammatory potential [30]. In the study carried out by Anand et al. [31], data on the concentrations of polyphenolic compounds in various plant sources are provided, with concentrations ranging between 15 and 30 mg·g^−1^. This indicates that the polyphenol concentrations obtained in our work are relatively high.

## 5. Conclusions

It can be concluded from this study that AgNPs produced through green synthesis using *H. illucens* biomass have antimicrobial capacity similar to AgNPs produced by chemical synthesis. This was demonstrated both by the inhibition of bacterial growth in CFU assay, and by the improved survival of *C. elegans* when infected by *P. aeruginosa*. Also, the presence of FUdR can influence the mortality of *C. elegans* when infected with *P. aeruginosa*. AgNPs from both syntheses prevented the death of chemically sterilized and non-chemically sterilized worms, protecting them from a *P. aeruginosa* infection.

## Data Availability

The original contributions presented in this study are included in the article. Further inquiries can be directed to the corresponding author.
